# Supplementary material for: Are carbon emissions trading and green financial instruments synergistic? -Comprehensive quantitative research based on content analysis
Source: PLoS One. 2024 Mar 7;19(3):e0298601. doi: 10.1371/journal.pone.0298601 (PMC10919601; doi:10.1371/journal.pone.0298601)
Supplement: S1 Data — (ZIP) [file pone.0298601.s001.zip › Data/地级市Z_F工作报告.zip!/╔╜╬≈/╜·╓╨╩╨/└┤╫╘╣1⁄2╓┌║┼í╛┬φ┐╦╩2╛▌═°í┐_2008.docx]

各位代表：

现在，我代表市政府向大会作报告，请予审议，并请各位政协委员和其他列席人员提出意见。

一、2007年政府工作回顾

2007年，在省委、省政府和市委的领导下，我们认真学习贯彻党的十七大精神，深入落实科学发展观，集中精力解决发展后劲问题，聚集财力解决民生问题，攻坚克难解决体制机制问题，下大力气解决发展环境问题，全市经济社会发展取得显著成效，圆满完成了各项目标任务。全年完成地区生产总值470.3亿元，增长14.7%；财政总收入88.06亿元，增长10.35%，剔除两权收入后78.16亿元，增长24.61%，一般预算收入（不含两权收入）31.12亿元，增长24.18%；规模以上工业增加值184.9亿元，增长23.3%；固定资产投资283.7亿元，增长40.9%；社会消费品零售总额162.1亿元，增长22.8%；城镇居民人均可支配收入10862.1元，增长18.2%；农民人均纯收入4206.7元，增长15.3%。

（一）经济结构持续优化，发展质量不断提高

三次产业协调发展。“三农”工作扎实推进，新农村建设规划逐步实施。粮食总产14.3亿公斤，创历史最高，蔬菜和肉蛋奶总产稳居全省前列；农产品加工、流通龙头企业销售收入46亿元，发展农民专业合作社1619个，占全省1/3以上；农产品“三品”认证74个,居全省第一；转移农村劳动力3.68万人；发展沼气用户6.7万户，是过去三年的6倍，居全省首位。工业经济势头强劲，工业效益综合指数达到149.1%，创历史新高。新增产值亿元以上企业31户，列入“四个梯队”的30户龙头企业销售收入增长44.3%，五大园区销售收入占规模以上工业近1/3；煤转电、煤转化进展顺利。第三产业素质增强，全年增速达到14.5%。旅游综合经济收入53.1亿元，相当于GDP的11.3%。对内对外贸易繁荣活跃，市场体系逐步完善，房地产、物流、金融等现代服务业发展势头良好。

资源环境继续改善。积极发展循环经济，工业循环经济园区有序建设，农业循环经济模式初步形成，生态示范村效益提高。认真落实节能降耗措施，淘汰落后工艺设备76台（座）、产能573.6万吨，单位工业增加值能耗下降9.87%，节能102万吨标煤。切实保护生态环境，关闭取缔污染企业665户，43家省控企业治理达标。主要污染物二氧化硫、化学需氧量分别下降7.33%和3.77%，圆满完成省下达任务。6个地表水断面治理成效显著，集中式饮用水源地水质达到国家标准。新造林36.92万亩。市城区绿化覆盖率、绿地率分别达到30.8%、26.2%，人均公园绿地7.5平方米。空气质量二级以上天数298天，增加53天，首次达到国家三级标准。灵石、介休、平遥、祁县退出全省空气质量监控重点县后十位行列。

科技创新能力有所提高。积极开展创新型企业试点、产学研技术联盟试点，设立了科技进步奖和科技担保基金，研究与试验发展经费支出占GDP的比重进一步提高，科技对经济增长的贡献率36%，高新技术企业增加值占地区生产总值比重6.3%。每十万人申报专利9.6项，提前完成“十一五”规划目标。

（二）重点工程扎实推进，发展后劲逐步增强

基础设施建设力度空前。全市新开工市政重点工程195项，完成投资24亿元，为前两年总和。市城区新开工8大类51项市政重点工程，完成投资6.6亿元，为历年最高。全市新增道路面积180万平方米，市城区集中供热率、供气普及率、污水处理率分别达到55.6%、87%、63%。实施水利重点工程116项，新建饮水安全工程220处， 100个行政村实现自来水标准化入户，新增和改善节水面积10万亩，新建和维修加固河道堤防107公里。汾邢高速晋中段开工建设，龙城高速前期工作进展顺利。新增公路通车里程586公里，县乡公路二级化改造254公里，占全省新增里程的47%。行政村全部通公路，村村通水泥（油）路3071公里，在全省领先。新增装机容量30万千瓦。新增35千伏以上变电线路258.1千米。

项目建设迈出较大步伐。引进项目114项，完成投资83亿元，合同引进资金298.8亿元，实际利用外资5749万美元，是上年的6.5倍。狠抓项目落地工作，办理立项手续210项，开工率76.6%。

体制机制日益完善。稳步推进国企改革，轻机厂和6户市直部门所属企业进入改制程序。深化财政管理体制改革，203个市级预算单位纳入国库集中支付。加强物价监测调控，市场秩序逐步好转。投融资体制改革、农村综合改革等各项改革均有突破，发展活力进一步释放。

发展环境不断优化。围绕规范、公开、效能三个重点，下大力气加强政务环境建设。新政务大厅建成运行，48个单位入驻，开展审批和服务事项409项。深化行政审批制度改革，精简审批项目263项，减幅55.5%。大力推行政务公开，不断扩大公开范围、拓宽公开渠道、提高公开质量。开通“百件实事网上办”专栏和政府12345便民服务热线。加强行政执法监督检查，严格实施“宁静生产日”制度。积极推进行政效能建设，努力提高行政效率和服务质量。坚持把改善金融生态环境作为政务环境建设的重要内容，依法清理国家公职人员和企业欠贷欠款1158万元，晋中商业银行组建运行，上海浦发银行正式落户。

（三）经济社会协调进步，发展内涵更加丰富

各类民生问题得到较好解决。新增城镇就业3.12万人，下岗失业人员再就业1.66万人，城镇登记失业率2.1％，零就业家庭基本消零。安置复员退伍军人408人。实行工资保证金制度，企业职工最低工资标准提高60元，企业养老、失业、医疗保险参保人数分别增加6400人、2400人和3.29万人。补签各类企业劳动用工合同3.75万份，追发拖欠工资598.78万元。城乡社会救助体系逐步完善，1424人享受城市医疗救助，为13.38万名城乡低保对象发放低保金7295.7万元，五保对象施保1.52万人。建设安康居住工程137.24万平方米，新建廉租住房7646.12平方米，棚户区改造28.11万平方米，采煤沉陷区新建住宅11.3万平方米，1338户群众迁入新居。2.17万人解决温饱，2.66万人摆脱贫困，扶贫移民1.09万人，解决了21.5万人饮水不安全问题。安全生产主要指标控制在省下达指标之内，各类伤亡事故起数和死亡人数分别下降23.7%和14.2%。

社会各项事业加快发展。教育经费保障机制改革顺利推进，全市财政教育支出增长22.87%，“两免一补”措施有效落实，201所农村寄宿制小学配置暖气，结束了火炉取暖历史。2个县通过省义务教育标准化验收,5所高中通过省示范高中验收。城乡医疗卫生服务体系逐步完善，新建社区卫生服务中心27个，新型农村合作医疗扩大到8个县(市)。推动文化晋中建设，成功举办第七届平遥(国际)摄影大展、晋中社火节等文化活动，非物质文化遗产保护继续加强。市体育馆建成使用。外事、侨务、气象、防震、档案、文物、地方志、民族、宗教、广电、新闻出版、人民防空、国防后备力量建设等工作取得新成绩，老龄、妇女、残疾人、人口和计划生育、关心下一代等事业都有新进步。

精神文明和民主法制建设不断加强。深入开展精神文明创建活动，加强未成年人思想道德建设，城乡文明程度进一步提高。各级政府自觉接受同级人大及其常委会的监督，积极支持政协开展工作，114件市人大代表建议和303件省市政协委员提案全部办结。积极开展法制宣传和法律援助，全民法制观念逐步增强。认真做好信访和人民调解工作，及时化解矛盾。严厉打击各类违法犯罪，加强社会治安综合治理，有力维护了社会稳定。强化行政监察和审计监督，政府自身建设得到加强，廉政建设取得新成效。

在回顾过去一年取得成绩的同时，我们也清醒地看到，我市经济社会发展仍然存在一定的差距和问题。主要是：经济发展质量还不高，经济总量还偏小，城市基础设施欠账较大，公共产品和公共服务与群众需求日益增长的矛盾还没有根本缓解，特别是全市仍然有4.58 万贫困人口没有解决温饱；发展中不协调的问题依然存在，三次产业协调带动能力不强，城乡一体化发展新格局形成速度不快，节能减排、环境保护和生态建设任务较重，提高城乡居民收入水平和生存质量存在较大压力；国有企业改革成本支付乏力，推进社会稳定与和谐发展任务艰巨；思想观念和行为方式与市场经济发展要求对接不够及时和紧密，创新意识不强，优化发展环境还需要一定的过程，尤其是各级政府部门和工作人员的服务水平、办事效率与群众的期望和要求还有不小的差距。我们一定要始终保持清醒头脑，增强忧患意识，以对晋中发展和人民福祉高度负责的使命感和紧迫感，更加兢兢业业、扎扎实实地做好政府各项工作，不辜负全市人民的信任和厚望！

各位代表，经过一年来的努力，全市“十一五”规划全面实施，经济社会发展整体推进，事关发展和群众利益的重大问题逐步解决，市政府向全市人民承诺的十件实事全部兑现！成绩来之不易。这是省委、省政府和市委正确领导的结果，是全市人民团结奋斗的结果。在此，我代表市政府，向全市工人、农民、知识分子、各级干部和离退休老同志，向大力支持政府工作的人大代表、政协委员、各民主党派、工商联、人民团体、无党派人士，向关心帮助晋中发展的国家、省驻地单位和驻地部队、武警官兵，向积极参与晋中建设的海内外朋友，表示衷心的感谢！

二、2008年政府工作的总体要求和奋斗目标

2008年是全面贯彻落实十七大精神的第一年，也是“十一五”承上启下的关键之年。做好今年的各项工作，我们面临良好的机遇：经济全球化和区域经济一体化进程进一步加快，生产要素和产业加速流动，有利于我们依托优势，承接东部地区和省城产业转移；国家确定山西为全国唯一的煤炭工业可持续发展试点省份，能源资源市场需求旺盛，煤炭市场预期良好，为我市煤焦等传统支柱产业稳定发展提供了有利条件；国家和省加大对服务业、煤化工、机械制造、农副产品加工和旅游业的扶持力度，新兴产业发展环境更加有利，我市区位、资源、产业优势将进一步发挥；中部崛起战略有利于我们获得更多的政策支持，大太原经济圈建设有利于我们拓展发展空间；县域经济实力整体提升，一大批重大项目带动效应逐渐显现，发展后劲不断积蓄，活力不断增强。同时，也面临全新挑战和巨大压力：受国家和省控制总量、淘汰落后政策影响，作为我市支柱产业的煤炭、焦炭行业迅速扩张难度加大；煤化工、电力、冶金等接续产业的行业准入门槛越来越高，发展难度和成本逐渐加大；国家进一步加大宏观调控力度，实施稳健的财政政策和适度从紧的货币政策，加大土地、环保、节能减排、项目审批、信贷投向等方面的刚性约束，我市扩大投资规模将更为艰难，投资拉动作用将受到一定制约。

各位代表，当前我市正处于经济总量快速扩张但发展压力不断加大、发展方式转变加快但增长约束明显增强、发展协调性有所改善但经济社会和谐要求不断提高的新阶段，面对发展的良好机遇和严峻挑战，我们一定要以只争朝夕的精神、奋勇争先的胆魄、富于创新的措施，自加压力，顽强拼搏，抓住机遇，奋力赶超，全面推进经济社会又好又快发展!

今年政府工作的总体要求是：高举中国特色社会主义伟大旗帜，以邓小平理论和“三个代表”重要思想为指导，深入贯彻党的十七大精神，全面落实科学发展观，按照市委二届三次全会的总体部署，围绕加快科学发展、构建和谐晋中这一主题，突出转变经济发展方式这一主线，以打基础、调结构、重民生、创环境、谋赶超为着力点，努力在调整产业结构、创新体制机制、建设生态文明、促进社会和谐、优化发展环境等方面取得突破，为建设山西中部最具活力的经济带和城市群奠定坚实基础。

主要预期目标是：地区生产总值增长12%；规模以上工业增加值增长18%；全社会固定资产投资增长24％；社会消费品零售总额增长15%；外贸进出口总额增长10%；财政总收入、一般预算收入分别增长17.7％、19％。

在努力完成以上指导性指标的同时，确保完成以下约束性指标：服务业增幅高于地区生产总值增幅；研发投入占全市生产总值的比例达到1%；单位生产总值综合能耗下降5.6%，二氧化硫、化学需氧量排放分别削减4.44%、9.24%；居民消费价格涨幅控制在4.7%以内；城镇居民人均可支配收入增长15%，农民人均纯收入增长8.5%；城镇登记失业率控制在4%以内；城市义务教育阶段免费教育全面实施；人口自然增长率控制在6‰以内。

完成上述目标任务，从我市发展的阶段特征、总体水平和强烈愿望出发，我们必须全力推进“四个发展”：

一要坚持科学发展。落实“三个转变”基本要求，加快转变经济发展方式，正确处理好与快、保与压、调控与发展的关系，把握政策机遇，克服矛盾制约，激活利好因素，促进产业转型升级，努力实现做大经济总量与提高发展质量相统一，加快发展速度与提高协调性相统一。

二要突出创新发展。以科技创新为核心，统筹推进思想观念、体制机制、发展环境和文化氛围创新，使创新成为发展的基本动力和干部群众的自觉行动，让各类要素的潜力竞相迸发，推动发展由资源依赖型向创新驱动型转变。

三要加快和谐发展。把构建和谐晋中摆在突出的战略位置，更加注重发展社会事业，更加注重解决民生问题，更加注重创造公平公正的社会环境，更加注重维护公共安全，更加注重生态文明建设，促进改革发展稳定相协调。

四要实施赶超发展。坚持好字优先、快字不忘，在追求好的同时，加快发展速度，各项经济社会发展指标该高的要高于全省平均水平，该低的要低于全省平均水平，缩小差距，负重爬坡，争先进位。

三、全力推进经济又好又快发展

（一）深化结构调整，加快构建晋中特色产业体系

着眼于转变经济发展方式和三次产业协调发展，夯实一产，主攻二产，大兴三产，推动传统产业新型化、新兴产业规模化，加快产业结构优化升级，逐步形成以农业为基础，工业和服务业双轮驱动，循环经济为主要经济技术模式，园区、企业和高新技术为支撑的具有晋中特色的产业体系。

强化工业主导地位，实施工业兴市战略，培育壮大工业支柱产业。围绕新型工业翻番目标，实施技术创新、项目带动和品牌战略，打造新型能源基地、煤化工基地、新型制造业基地三大板块，整体提升工业经济规模和效益，增强工业带动能力。机械制造业要壮大纺机、液压、汽车改装、铸锻件等产业集群，抓好山西鸿基纺机、寿阳金牛煤机等重点项目建设；化工行业要加快发展以焦炉煤气利用、焦油加工为基础的煤化工工业，重点抓好榆化双40万吨烧碱及PVC、三佳6万吨有机硅、中煤九鑫30万吨甲醇、路鑫30万吨粗苯精制等项目；电力产业要积极推进大型燃煤机组、热电联供机组和资源综合利用机组建设，加快榆次国电、山西瑞光等电源项目建设；冶金行业要重点抓好安泰集团120万吨H型钢和100万吨高速线材、山西三明重工30万吨不锈钢无缝钢管等项目；煤炭工业要加大资源整合、重组和兼并力度，引深与阳煤集团的战略合作，抓好寿阳新元600万吨、昔阳寺家庄600万吨等重点矿井建设，加快打造地方煤炭集团“航母”；焦化产业要上大关小，提高产能，延伸产业链条；医药行业要扶持发展山西医药工业园、太谷医药工业园和榆社医药工业园三大园区，重点抓好中远威5亿片溶栓片生产线、500万支眼药水、山西通江700吨纳米消毒材料；食品加工业要加快建设平川农副产品深加工食品基地和太行山高寒山区绿色农产品加工基地，培育平遥牛肉、食醋、小杂粮、果品四个特色加工集群，加快发展乳制品、饮料酒、干鲜果菜加工，重点抓好娃哈哈二期、平遥牛肉2万吨肉制品、通宝醋业5万吨纯净醋等项目；轻纺工业要加大技术创新力度，大力发展精深加工，重点抓好山西大华钡冕玻璃器皿生产线、山西晶鹏玻璃器皿深加工、山西恒天700吨碳纤维原丝等项目。

突出发展服务业，加快形成工业和服务业双主导发展格局。推动资源优势向产业优势、市场优势和经济优势转变，改造提升传统服务业，加快发展新兴服务业，全面提高服务业规模和水平，服务业占GDP比重提高1个百分点。一要做大旅游产业。整合盘活旅游资源，抓好太谷梅苑生态园、榆次乌金山清泰安养园、祁县乔家大院等一批景区景点建设项目。加强景点景区住宿餐饮、道路交通、环保卫生、供水供电等基础设施建设，重点在榆次、平遥、介休、灵石等地新建一批星级旅游饭店。抓住奥运会“赛在北京、游在山西”的机遇，以平遥国际摄影大展、晋中社火节等节庆活动为龙头，打好促销组合拳，大力推介旅游精品，打响晋商、大寨两大品牌。推进无障碍旅游区建设，开通旅游“绿色通道”，整治旅游秩序，优化旅游环境，提高服务水平，积极创建国家旅游服务标准化示范城市。以建设山西旅游服务集散中心为目标，围绕太原晋中同城化发展，整合旅游资源，加大旅游合作，鼓励组建跨行业、跨部门、跨所有制、跨区域的企业集团，实现规模化经营，构建大旅游格局。二要积极发展现代物流业。依托榆次鸣李交通运输交汇点的优势,规划建设综合物流园区,加快把中心城区建成全省物流核心枢纽。积极建设介休煤焦化专业物流中心。三要加快发展房地产业。规范房地产市场，加快房地产开发，统筹推进学校、医院、购物、绿化、道路管网、交通通讯等功能性配套设施建设。坚持保护与开发并举，抓住旧城改造和新城开发契机，投资50亿元，开发房地产面积420万平方米。四要推动现代金融业发展。扩大国有商业银行服务范围，增加资金运用总量，开展金融机构执行货币政策情况评估。加大“招行引资”力度，积极引进兴业、中信、民生等股份制银行。大力发展地方金融，做好城市商业银行在县市设立分支机构工作，推进农村信用社改革，提高服务水平。加强政策性担保体系建设，鼓励发展商业性担保公司和多种形式的担保机构。积极发展商业性小额信贷公司。继续清理国家公职人员和企业欠贷欠款。

大力发展园区经济，促进产业集聚发展。坚持产业集聚、行业集中、土地集约、管理集成原则，落实规划高起点、基础高标准、产业高配套、投资高密度要求，加快市级工业园区和县级特色园区建设，引导企业和项目向园区集中。坚持节约集约用地，严格工业园区投资强度最低标准，鼓励企业在原有土地上进行增资扩股或“二次招商”。晋中开发区要加快形成产业集群、规模集约、功能明晰、设施配套的高标准工业聚集区，努力打造晋中工业“小硅谷”。加快榆次工业园、安泰工业园、中煤九鑫工业园、榆社化工园、平遥工业新区、祁县经济开发区等园区发展，园区销售收入力争达到全市规模以上工业总量的三分之二以上。

实施项目带动，培育发展一批大企业大集团。坚持大开放、大引进、大投入，落实项目建设跟踪服务制，集中建设60个工业重点项目，特别在电力、煤化工和汽车改装项目建设上要有重大突破。积极推进技改、联合、嫁接、重组、上市，加快企业资金、技术和人力资源整合，培育和发展一批技术进步、主业集中、比较优势和规模优势突出的大型企业集团。推动资源配置向梯队企业集聚，土地供应向梯队企业倾斜，产能和环境容量指标向梯队企业集中，不断提升产业集中度。加快企业家队伍建设，发挥企业家引领带动作用。

发展现代农业，夯实农业基础，增强农村经济活力。按照中央“三个明显高于”的要求，不断加大农业投入。认真落实国家、省扶持农业和粮食生产“十项政策”，稳定粮食生产，提高粮食综合生产能力。优化农产品布局，扩大优势农产品基地，启动建设“金三角”现代农业示范区，培育无公害蔬菜、特色干鲜果和健康养殖业基地，发展一批标准化生产示范园区、规模养殖示范小区和示范村。积极发展生态农业、观光农业。高标准实施农业产业化经营，发展壮大龙海公司等一批龙头企业，销售收入与带动农户数均增长11％以上。建设区域性大型农产品综合批发市场，完善市县乡村四级市场体系，提高农产品市场占有率,新建农家便利店300个。加大农民专业合作社“一建两改”力度，推进合作社之间，合作社与农村信用社、供销社互动发展。实行农产品市场准入制度，建设市级农畜产品质检中心。切实抓好农产品标准化生产，大力发展无公害农产品、绿色食品和有机农产品，不断优化农产品品质结构，提高农产品质量标准和市场竞争力。加快农业机械化进程。实施“十万农民网上行”工程。推开“农民增收明白卡”制度试点工作。

（二）加快转变发展方式，推动经济永续发展

充分发挥科技创新第一推动力的作用。完善多元化投入机制，建立科技创新担保资金，形成3000万元科技贷款规模。以企业为主体、市场为导向，引导和支持技术要素向企业集聚，不断提升企业的技术承接和创新能力，促进科技成果向现实生产力转变。指导规模以上企业建立技术研发机构，启动建立纺机、液压、煤化工、新材料、医药等技术中心和工程研究中心。出台促进产学研合作的具体意见，创立“晋中本土”院校、科研机构的产学研合作平台，多层次开展科技培训，多渠道引进科技人才，逐步建立区域科技创新体系和人才创新机制。实施农民素质提升工程，开展农民科技培训100万人次。

提高消费对经济的拉动作用。正确处理投资和消费的关系，提高城乡居民的消费能力，努力培育住房、汽车、健康、旅游等消费热点。优化城区、景区商贸网点布局，提高企业经营档次和服务质量，满足即期消费，激活潜在消费，创造未来消费。加快田森等连锁企业改造升级和规模扩张，积极引进国内外知名流通企业。实施零散小商铺升级改造，纳入便利店管理网络。积极开拓农村市场，发挥供销合作社和各类专业合作社的网络作用。强化对生活必需品等重要商品的市场监管和调控，建立防范市场异常波动应急机制。

全面发展循环经济。加快编制工业循环经济发展规划，深入开展循环经济县、园区、企业创建活动。抓好介休市、榆次东阳镇、安泰、三佳等全省循环经济试点启动和推进工作，积极创建生态工业园区。加快推进清洁生产，在全市93户重点企业中强制实行清洁生产审核。搞好环境管理体系认证。

不遗余力推进节能减排。以煤炭、焦炭、电力、化工、冶金、建材六大行业为重点，继续推进“百企节能行动”，推广能源合同管理，建立能源计量、统计制度，落实能源审计、节能规划等措施，实施重点监控，促进节能技改项目尽快落地见效，全年节能80万吨标煤。强化属地管理，淘汰落后企业16户、产能193.2万吨。开展固定资产投资项目节能评估和审查，提高准入门槛，管住用能增量，坚决否决不符合节能要求的新上项目。加大非煤产业资源整合力度，鼓励引导煤炭企业进入一、三产业领域，加快资源型企业转产、转型。加强矿产资源综合开发利用、再生资源回收利用和生活垃圾资源化利用，不断提高资源利用水平。完善节能减排责任制，严格问责制和一票否决制。

加强生态建设和环境保护。认真实施国家天然林保护、退耕还林、太行山绿化和省级造林绿化工程，推进全市“双十一”造林绿化工程，造林38万亩，植树800万株以上，实有林木面积率增长1.2%。深化林权制度改革，推进营林机制创新。坚持依法治林，加强林木管护，分阶段全部封山禁牧。全面实施蓝天碧水工程，大力治理工业污染，实行环境容量总量控制，落实环境影响评价和“三同时”制度，强化新建项目和企业运行全程监管，工业企业全部配套污染防治设施，实现污染物达标排放。加强采矿区环境治理和生态恢复。大力整治市城区燃煤锅炉、扬尘污染源和机动车尾气污染，城市集中供热管网覆盖区域9月底全部入网。集中精力搞好汾河清水复流工作，祁县、平遥、介休、灵石重点抓好主河槽加固和河道岁修工程，加快疏浚整治河道河床。联合打击河道内人为设障和采砂行为，切实做好汾河干流的水质保障和水环境监测等工作，确保汾河长流常清。

各位代表，生态建设和环境保护是建设生态文明的重要内容，是事关晋中对外形象和投资环境，事关人民生活质量和健康水平的重大任务！我们一定要坚持生态优先、协调发展，打破以资源环境为代价的传统发展模式，告别一切与生态文明不协调的行为习惯，把生态建设和环境保护落实到每个单位、每个家庭、每个角落，让生态文明观念深入人心，全力建设一个资源节约、环境友好的生态晋中！

（三）加强城乡建设管理，提升城市承载能力

按照“四城联创”总体部署，引深基础设施建设年活动，推动城乡建设管理上水平，构建城乡一体化发展新格局。

城市规划、建设和管理力求突破。高标准完成《城市综合交通规划》、《部分主干道街景规划》、《城市规划年度实施计划》编制，积极推进《城市总体规划》修编工作。坚持质量第一，抓好汇通路南出口、环城东路等路网改扩建工程，思凤街立交桥、龙湖立交桥等桥梁新建续建工程，新华街片区、顺城西街片区等5项城中村及城市棚户区改造工程。抓好供水、供热、排水、垃圾处理、污水处理等事关民生的基础设施建设，全市集中供热普及率达到52.5%，燃气普及率达到72%。加快汾邢高速建设进度，开工建设龙城高速，构建环城和区域两个高速公路循环圈。完成国省干线改造。加强电网工程建设，新增供电容量90万千伏安。巩固完善防洪、防震等设施。推进管理下沉，逐步建立统一领导、各司其职、规范管理、强化基层的管理机制。依托网格化、数字化等先进技术手段，探索城市数字化管理新模式。

大力发展特色城镇。坚持市区、县城、集镇、工业园区并举建设、多元发展，加快形成优势明显、布局合理、各具特色、辐射强劲的城镇群。根据产业特色和自然条件，加大投入，统筹规划，促进城镇区域规划与工业园区及商贸、交通、教育、文化等社会事业规划有机配套，互相照应，形成联动效应。城镇化率提高2个百分点。

统筹城乡发展，加快新农村建设。完善农村社会化服务体系，构建以工补农、以城带乡的全新机制。突出抓好农业和农村基础设施建设,继续落实“五清五规范”、“四化四改”、“十有十个一”任务，引深“创三优”活动。大力实施兴水战略，加快新水源开发、水库和水电站建设以及中小型水库除险加固，新建集雨旱井1000眼，维修改造小型水利工程100处，新增和改善节水灌溉面积10万亩，治理河道60公里。加快农村公路建设，新增公路通车里程200公里，县乡公路二级化改造260公里，新建乡镇汽车站9个，继续推进城乡客运一体化。大力改善农村能源结构，努力转变农村生产生活方式。加大扶贫开发力度，认真落实“两区”建设项目。加强以劳动用工、社会治安、安全生产等为主要内容的农村基础管理，维护农村稳定。

全方位、大力度开展城乡环境综合整治。重点对城市出入口、居民小区、背街小巷、河流沟渠、农贸市场、城中村和农村地区等重点区域和薄弱环节进行重点整治。综合推进城中村改造，切实解决城中村垃圾处理、污水排放等问题。规划建设公益性交易市场，规范集贸市场，引摊进场入市，严厉打击“伸舌头”经营。创建一批园林化单位和园林化小区，搞好市城区9条道路绿化和部分小街巷绿化，人均公园绿地新增2平方米，绿化覆盖率和绿地率分别达到35.3%、30.2%。打通一批“断头路”，改造一批“瓶颈路”，增强城市主干道和快速路的通行能力，提高分支道路、小街小巷利用率。以街巷硬化、村庄绿化、环境净化、路灯亮化为标准，开展农村环境整洁行动，彻底改变脏乱差。

（四）创新体制机制，为发展提供持久动力和坚强支撑

着力破解制约改革发展的深层次矛盾和问题，努力在一些重点领域和关键环节取得重大突破，加快形成促进改革发展的动力机制和支撑体系。

全面推进各项改革。加快国有资产管理体制改革和国有经济结构布局调整，建立完善国有资产出资人制度。坚持以稳定为前提，以清晰产权为核心，以置换职工身份为重点，深入推进国企改革，晋华厂、锦纶厂、轻机厂争取完成政策性破产，加快推进晋中建设集团、金恒化工、晋中药材公司和市直部门所属企业改制工作。深化公共财政改革，调整优化支出结构，重点支持公共事业发展。强化预算管理和监督，提高财政资金使用效益。加强政府采购监督管理，构建“阳光采购”。落实资源税费改革政策，完善资源有偿使用制度和生态环境补偿机制。加快事业单位人事、分配、产权等各项制度改革，建立政事职责分开、单位自主用人、人员自主择业、政府依法管理、配套措施完善的分类管理体制。

拓展对外开放的广度和深度。组织好各类招商活动，突出主动招商，力争引进项目70个以上，完成投资100亿元以上。加快落地项目建设进度，提高外资实际到位率。强化企业招商主体作用，鼓励企业参加各类经贸交流活动。注重在存量建设用地和未利用土地上挖掘潜力，缓解项目建设用地矛盾，促进项目顺利实施。根据区位、资源、产业特色，主动融入大太原经济圈，加快区域一体化发展进程。大力营造开放氛围，让开放成为我们的思维方式和行为习惯，为发展注入更加强大的动力和活力！

大力推进人才强市。加快建立健全人才开发资金投入机制和人才政策体系，抓住培养、吸引、使用三个环节，统筹高层次人才和高技能人才为重点的人才队伍建设。大力开展招才引智，畅通人才引进 “绿色通道”，积极引进各类人才。建立面向全社会的人才管理制度，加快形成人尽其才、才尽其用、充满生机的人才发展环境和尊重劳动、尊重知识、尊重人才、尊重创造的氛围，激发各类人才的创造活力和创业热情，为经济社会发展提供有力的人才保证和智力支持。

四、加快构建和谐晋中

全面推进以改善民生为重点的社会建设，切实解决群众生产生活问题，大力推进公共服务均等化，促进社会公平正义、繁荣发展、和谐稳定。

（一）认真实施“五大惠民工程”

抓好教育协调发展工程。完善义务教育经费保障机制，继续推进学校标准化建设。深化教育人事制度改革，培养一批名师名校长、省市学科带头人和骨干教师。加强农村教师队伍建设，注重发展学前教育，推进城乡教育均衡发展。加快市县乡校四级信息网络建设。率先在全省基本普及高中阶段教育。大力发展职业教育，每县至少办好一所示范性中等职业学校，以晋中职业技术学院为龙头，组建区域性职教集团和职工培训及技能鉴定协会，加强校企合作、工学合作，争创全省一流水平。扎实做好贫困家庭、进城务工农民子女和农村留守儿童的教育工作，落实减免、补助措施，确保每个孩子都有学上、上得起。抓好创业就业工程。加快建立以公共职业介绍机构为主体、覆盖城乡、功能完备的创业就业服务体系，完善困难群众就业援助制度，有效增加就业岗位。抓好医疗健康工程。大力发展农村卫生、公共卫生和社区卫生，加强城乡医疗卫生基础建设，深化医疗卫生体制改革，规范医疗服务行为，继续开展县级以上医疗机构药品网上集中竞价采购，乡村医疗机构和社区卫生服务中心实行药品集中询价采购，努力为群众提供安全、有效、方便、价廉的医疗卫生服务。抓好社会保障工程。全面推行城镇居民医疗保险，力争将失地农民全部纳入社会保障和城市公共就业服务范围，机关事业单位职工全部纳入生育保险，实行工伤保险市级统筹。扩大非公经济组织社会保险覆盖面，妥善解决市属国有关闭破产企业退休人员和国有特困企业职工参加医疗保险问题。完善城乡社会救助体系，城乡低保应保尽保，五保供养标准达到当地居民人均生活水平，集中供养率达到12%。加快建设市、县儿童福利院和流浪未成年人救助中心。抓好住房安居工程。多渠道解决群众住房困难，加快中低价位、中小型普通住房建设，健全廉租住房保障制度。要进一步加强测评考核、加强基层基础工作、加大财政投入力度、实行干部下访等措施，保障和推进 “五大惠民工程”有效落实。

各位代表，办好民生大事，是人民政府义不容辞的责任。在扎实做好各项工作、全面完成省政府提出的十件实事的同时，今年市政府要在改善群众生产、生活方面着力办好十件实事：一是新建、改建、扩建50所中小学，努力解决城镇中小学班容量过大问题。二是千方百计扩大就业，创业培训2000人，创业就业6400人；城镇新增就业2.88万人，困难对象再就业3240人，零就业家庭实现动态消零；完成农村劳动力技能就业培训1.2万人，劳动力转移引导性培训6万人；转移农村富余劳动力3万人。三是新型农村合作医疗制度实现全覆盖；启动城镇居民医疗保险，参保率达到50%；完成458所村卫生室的达标建设，县乡村三级医疗卫生机构基础设施80%达标；新建5所和完善27所社区卫生服务中心，新建城市社区卫生服务站20个，城市社区卫生服务机构建设完成规划任务的90％以上。四是新建经济适用住房25万平方米，解决4166户城市中低收入家庭的住房困难；新建廉租住房8.58万平方米，解决4600户廉租住房保障对象的住房困难；扶贫移民搬迁1万人；集中治理34个地质灾害严重村，解决4244户、13954名农民的住房安全问题。五是市城区和介休市的生活垃圾厂基本建成，其它县的生活垃圾处理厂完成前期并开工；市城区第二污水处理厂和太谷污水处理厂完成主体工程，介休、祁县、和顺、寿阳、灵石污水处理厂建成并投入使用，平遥、昔阳、榆社、左权污水处理厂完善设施并保证正常运行；市城区二级以上天数达到290天以上，东山5县均达到252天，平川5县（市）均达到240天。六是解决21万人的饮水不安全问题，完成100个行政村自来水入户工程。七是实施农村新能源工程，发展沼气用户4万户。八是扶持发展农民专业合作社，争取新增1000个，重点扶持500个，培育示范合作社100个。九是全市完成70条小街巷改造，其中市城区20条；市城区30条次干道、小街巷安装路灯。十是村村通水泥（油）路和新农村街道硬化1500公里，开工率、合格率、完工率均达100%。

（二）加强和完善社会管理

健全党委领导、政府负责、社会协同、公众参与的社会管理格局，坚持以人为本，创新社会管理理念和管理方式。深入开展迎奥运、创平安、保稳定、促发展活动，加大防范工作力度，落实综合治理措施。严格落实信访责任制，畅通社情民意反映渠道，解决群众反映强烈的热点难点问题。完善经常性矛盾纠纷排查调处机制，掌握苗头性和倾向性问题，及时化解矛盾。建立健全统一指挥、协调有序、反应灵敏、运转高效的突发性、群体性事件处置机制，提高处理各类群体性事件和突发事件的能力。加强人口和计划生育工作，稳定低生育水平。落实民族宗教政策，依法管理民族宗教事务。继续做好档案、气象、地震、人防、地方志、国防后备力量建设、老龄、双拥、侨务、残疾人等各项工作。

（三）切实抓好安全生产

严格安全生产责任，完善安全生产管理体制和监管机制，建立安全生产应急救援体系，提高防范应对事故灾难的能力。深入开展安全隐患治理年活动，推进乡镇安全监管机构建设，加大监管力度，提高监管能力，坚决防止重特大事故发生。突出抓好煤矿安全生产，重拳出击，严厉打击私挖滥采、超层越界开采、超能力、超强度、超定员开采等非法违法行为。坚持“抓综治、保畅通、保安全、为人民”的要求，突出源头治理，重在长效管理，无缝隙、拉网式集中治理车辆超限超载。巩固扩大产品质量和食品安全专项整治成果，严格执行生产许可、强制认证、注册备案制度，严把市场准入关，提高涉及人身健康和安全产品的生产许可条件和市场准入门槛。加强道路交通、危险化学品、民爆物品、文物消防、动物疫病等重点领域的安全管理。 各位代表，抓安全就是促发展、就是保稳定，只有重视安全生产，才能推进安全发展，只有落实安全措施，才能实现赶超目标。我们必须时刻绷紧安全这根弦，三百六十五天天天不忘、紧抓不放，为又好又快发展和人民安居乐业提供坚强保障！

（四）推动文化大发展大繁荣

积极发展文化产业。继续推进文化体制改革，重塑文化市场主体，进一步解放和发展文化生产力。加快建设文化产业园区、基地、企业，扶持一批以文化经营为主体的企业集团，打造一批富有晋中特色、具有一流竞争力的文化精品。促进文化产业与旅游、体育、教育、科技、信息等行业联动发展，构建文化产业发展新格局，提高文化产业对经济发展贡献的比重。扶持发展民办文化，加强网络文化建设和管理，继续办好各类文化节庆活动。

强化公共文化服务。加大投入力度，积极构建覆盖全社会的公共文化服务体系，加快公益性文化事业发展。推进农村电话、电视、互联网“三网合一”，加快村级网络文化站建设。进一步提高县级图书馆办馆标准，加强基层文化馆(站)建设。做好非物质文化遗产的重点普查和全省第二批非物质文化遗产项目申报工作。加强文物保护，加快市博物馆筹建工作，做好第三次全国文物普查工作。强化传承传统文化意识，营造文化发展氛围，为三晋文化繁荣发展做出贡献。

（五）加强民主政治和精神文明建设

推进社会主义民主政治建设。各级政府要定期向人大及其常委会报告工作，自觉接受政协的民主监督，认真听取民主党派、工商联、无党派人士和各人民团体的意见，积极办理人大代表建议和政协委员提案，支持政协开展工作，主动接受社会监督和舆论监督。加强基层民主建设，努力扩大政务公开、村务公开和厂务公开的范围和内容，保障人民群众的选举权、知情权、参与权和监督权。

加快依法治市进程。认真贯彻“五五”普法规划和依法治市纲要，全面落实行政执法责任制，健全政府依法行政制度，强化行政执法监督，推进行政行为的程序化、规范化、法制化。加强社会治安综合治理，深入开展平安创建活动，依法防范和打击违法犯罪活动，确保社会安全稳定和人民群众安居乐业。加强市场监管，深入整顿和规范市场经济秩序，保障各类市场主体合法经营，促进市场经济健康发展。搞好法律援助工作，维护妇女、儿童、残疾人和社会弱势群体的合法权益。深入开展法制宣传教育，在全社会形成崇尚法制、遵守法律、依法办事的良好氛围。

加强社会主义精神文明建设。强化理想信念教育，在全社会树立中国特色社会主义的共同理想。深入开展社会主义荣辱观教育，建立完善社会公德、职业道德、家庭美德和个人品德等思想道德体系，在全社会形成爱国守法、明礼诚信、团结友善、勤俭自强、敬业奉献的基本道德规范。深入开展城乡文明和谐创建活动，不断提高城乡文明程度。

五、全面加强服务型政府建设

创新政府管理理念、管理体制和管理方式，改进作风，狠抓落实，努力建设服务型政府。

一要推进改革创新，提升政府创新力。继续解放思想，转变观念，敢于打破陈规陋习、打破条条框框，注重用市场经济的规律、用改革开放的办法思考和解决问题。保持奋发有为的精神状态，勇于谋大事、干大事、创大业，善于抓关键、攻难点、促全局，自我加压、主动工作。充分调动各方面的积极性、主动性、创造性，努力形成艰苦创业、开拓创新、争创一流的生动局面。

二要加强效能建设，提升政府执行力。突出“快”字，发扬雷厉风行的作风，继续加快电子政务建设，完善面向基层、面向群众、高效快捷、运转顺畅的工作机制，形成高效率、快节奏的优良政风。突出“闯”字，敢于面对风险，不怕担责任；敢于面对困难，不怕吃苦头；敢于面对矛盾，不怕得罪人，破解执行障碍，创造性地开展工作。突出“实”字，大兴求真务实之风，提倡少开会、开短会、少讲话、讲短话。突出 “严”字，强化目标责任制，健全监督机制，落实督查措施，确保政令畅通、令行禁止。

三要坚持执政为民，提升政府公信力。自觉把维护人民利益作为政府决策的首要依据，以群众是否满意、是否高兴、是否拥护作为工作的根本标准，多做群众急需、“雪中送炭”的事，多做顺应民心、群众受益的事，多做打基础、管长远的事。抓好市县乡村四级政务服务平台建设，为群众和投资者提供快捷、便利、优质服务。严格落实行政不作为问责制和过错责任追究制，严肃处理不作为、慢作为、乱作为的人和事，对吃拿卡要者形成“触电身亡”的高压之势。坚持诚实守诺，取信于民，带头加强信用体系建设，做到说一句，是一句，句句算数；干一件，成一件，件件落实!

四要严格依法行政，提升政府约束力。认真贯彻《行政许可法》、《公务员法》等法律法规，严格按照法定权限和程序行使职权、履行职责，做到有权必有责、用权受监督、违法受追究、侵权须赔偿。完善《政府工作规则》，健全领导、专家、群众相结合的决策机制，落实决策咨询、听证、公示制度，进一步规范政务公开的内容、方式和程序。

扎实推进反腐倡廉建设，全面落实政府系统党风廉政建设责任制，建立健全教育、制度、监督并重的惩治和预防腐败体系。规范土地和矿产资源开发、政府采购等公共资源管理。加大专项治理力度，重点解决环境保护、食品药品安全、安全生产、土地征用和房屋拆迁等群众反映强烈的问题，坚决纠正损害群众利益的不正之风。大力提倡艰苦奋斗，坚决制止奢侈浪费。加强对领导干部、人财物管理使用、关键岗位的监督和制约，清清白白、干干净净为人民办实事、谋利益。

五要保持团结和谐，提升政府凝聚力。善于从全局的高度认识问题、统一思想，各级政府班子都要做到在政治上同德、事业上同心、工作上同力。坚持民主集中制原则，既要防止和克服独断专行、搞家长制，又要防止和克服软弱涣散、各行其是。广大公务员都要严于律己，宽以待人，相互尊重，相互支持，形成能够担负历史使命、团结带领广大群众更好更快发展的坚强有力的战斗集体。

各位代表，创造一个经济更发展、社会更和谐、生态更文明、人民更幸福的良好环境，是建设服务型政府的基本任务。今年，要在全市深入开展“环境建设年”活动，努力在六个方面建设一流环境。一是以行政审批制度改革、推进政务公开、严格行政执法、优化政务服务为主要内容，努力建设一流的政务环境；二是以建设社会信用体系、开展金融综合服务、发展资本市场为主要内容，努力建设一流的金融生态环境；三是以坚持依法行政、促进司法公正、维护公共利益为主要内容，努力建设一流的法制环境；四是以“保洁”、“治乱”、“促秀”、“畅通”为主要内容，努力建设一流的城乡基础环境；五是以加强安全生产、维护公共安全和社会稳定为主要内容，努力建设一流的安全环境；六是以节能降耗减排、蓝天碧水工程、造林绿化为主要内容，努力建设一流的生态环境。

各位代表，晋中是我们共同的家园，晋中的发展与每一位晋中人都息息相关，晋中的美好未来也要靠全市人民共同创造。作为当代晋中人，只要我们继承和发扬以自力更生、艰苦奋斗为灵魂的大寨精神，不怕牺牲、不畏艰险、敢于胜利、无私奉献的太行精神，节俭勤奋、明礼诚信、精于管理、勇于开拓的晋商精神，大力倡导科学发展、以人为本、与时俱进、开拓创新的时代精神，就一定能够实现赶超发展的新目标，做出无愧于时代和人民的新业绩!

各位代表，奋力赶超、争先争上，把晋中的明天建设得更加美好，是时代赋予我们的神圣使命。让我们在省委、省政府和市委的坚强领导下，全面贯彻落实“三个代表”重要思想和科学发展观，开拓创新、求真务实、负重拼搏、埋头苦干，为实现赶超发展、早日把晋中建设成为山西中部最具活力的经济带和城市群而努力奋斗！
